# Supplementary material for: Keep your budget together! Investigating determinants on risky decision-making about losses
Source: PLoS One. 2022 Mar 21;17(3):e0265822. doi: 10.1371/journal.pone.0265822 (PMC8936482; doi:10.1371/journal.pone.0265822)
Supplement: S1 Appendix — (PDF) [file pone.0265822.s002.pdf]

S1 Appendix

Apparatus in detail

Stimulus presentation and response registration were controlled by one of six computer systems. Two of them were running a 64bit Linux OS (Linux Mint v. 17 and Linux Ubuntu v. 12.10) with a standard 22" LCD wide-screen monitor (screen resolution: 1920×1080 pixels). The other four computer systems were running a Windows 7 64bit OS, two with a standard 22" LCD wide-screen monitor and two with a standard 17" LCD monitor (screen resolution: 1280×960 pixels). The control software operated on Matlab® 2014a including the Psychophysics Toolbox version 3.0.13 [42,43]. The input device was a USB-controller with four buttons in a row, with the two center buttons being disabled. The participants were encouraged to press the left button with the index finger of the left hand and the right button with the index finger of the right hand.

The monitor display had a white background; the instruction text there was written in black with font "Calibri". The main font size of the text was 24pt for the 22" LCD and 16pt for the 17" LCD. Choice options were represented as two pie charts displayed next to each other in the center of the screen. One pie chart represented the sure option (filled circle with light gray indicating loss-frames or dark gray indicating gain-frames; the number of the amount initially taken was displayed in the center of the circle). The other pie chart represented the gamble (probabilities of the risky option were indicated by the respective areas in the pie chart, with dark gray indicating the probability of winning the gamble and light gray indicating the probability of losing the gamble). The numbers of expected gamble outcomes were added to the areas; text there was written in white. An example of the display is shown in Figure 1 C (gain frame) and Figure 1 D (loss frame). The diameter of the pie charts representing the choice options was 250px. The sure or the gamble option was randomly presented on the left or the right side. Available time for making a decision was indicated by eight vertical bars displayed at the bottom of the screen. The bars disappeared one by one as a function of the preset time limits, i. e., the

given time limit divided by eight (see Figure 2). Note that participants knew the total number of trials per block (i. e., the length of one period). However, they received no information about the current trial number, i. e., they had to count the trials themselves or estimate the number of the remaining trials.
