# Supplementary material for: Keep your budget together! Investigating determinants on risky decision-making about losses
Source: PLoS One. 2022 Mar 21;17(3):e0265822. doi: 10.1371/journal.pone.0265822 (PMC8936482; doi:10.1371/journal.pone.0265822)
Supplement: S2 Appendix — (PDF) [file pone.0265822.s003.pdf]

S2 Appendix

T-tests: preference changes

Table 2

Preference changes between the need conditions for the current study and for the study by Diederich et al. (2020).

	Decisions about losses			Decisions about gains		
	t-value	df	p-value	t-value	df	p-value
Need 2,800 vs. Need 3,600	.048	83	.962	−.237	105	.813

Table 3

Preference changes between the current study and Diederich et al. (2020) for each need condition.

	Need 2,800			Need 3,600		
	t-value	df	p-value	t-value	df	p-value
Decisions about losses vs. gains	.467	93	.641	.196	95	.845
