# Supplementary material for: Keep your budget together! Investigating determinants on risky decision-making about losses
Source: PLoS One. 2022 Mar 21;17(3):e0265822. doi: 10.1371/journal.pone.0265822 (PMC8936482; doi:10.1371/journal.pone.0265822)
Supplement: Attachment — Submitted filename: Response_to_Reviewers.pdf [file pone.0265822.s004.pdf]

Dear Dr. Worthy, dear reviewers,

We are very grateful for a constructive and thoughtful set of reviews, which have substantially improved the paper, and the opportunity to resubmit a revised version of our manuscript.

We append a detailed record of all the changes below.

Response to Editor and Reviewer
(Replies are in red and start with *)

Editor comments:

One thing that I will specifically require for a revision is either an increase in the sample size, or a replication study. Based on the reviews and my reading of the manuscript, I don't think it will be possible to draw meaningful conclusions from the data without additional data being collected.
[***Editor comment from a later correspondence***: (…) If you think you can successfully convince the reviewers that the sample size is adequate then I will not make it a firm requirement to increase the sample size. If you decide to not increase the sample size, then please try to explain why the sample size is appropriate in your response to reviewer comments. You can refer to this communication if you think that will be helpful, so that reviewers know that I said it was not firm requirement if they agree that the approach is appropriate.]
*In our study, we used a psychophysical data-collection approach, which is relatively new to this research field. Instead of presenting few trials to many participants as it is typically done in social science, fewer participants perform many more trials here, a common approach in cognitive science. In the context of framing in risky decision making, it had been used by, for instance, De Martino et al., 2006 (Science), Guo et al., 2017 (Psychological Science), Diederich & Trueblood, 2018 (Psychological Review), Diederich et al., 2020 (Theory and Decision), Wyszynski et al., 2020 (PLOS ONE), Roberts et al., 2021 (Psychological Science).
Furthermore, as commonly practiced, we determined the sample size according to a power analysis based on 1.) standard parameter of alpha=0.05, and a required power of 0.8, which is above standard 2.) the smallest significant effect size (interaction effect; Frame x Need (3600)) reported in a previous study (Diederich et al., 2020), and 3.) the statistical test we used for the analysis: linear mixed model with "participants" and "trial numbers" (within one block) as grouping factors (random effects). We used the R package SIMR that allows calculating power for generalized linear mixed models.
Nonetheless, we increased the required power for the smallest effect size to 0.9 by increasing the sample size to n=40 participants. Note that the power of the main effects, tends towards 1.

Reviewer #1 comments:

The time limits are very strict. As a result, in "catch" trials, the dramatically less attractive option was chosen as often as 25% of the time.
* Previous studies using identical time constraints in very similar designs reported higher catch trial accuracy (83% Gou et al. 2017; 89% Diederich et al. 2018). Consequently, we do not assume a direct relationship between the length of the time limits and catch trial accuracy.

More generally, it is not clear why we should be interested in time limits here. If this is an attempt to test Stephens' model, it seems misguided because we only have one interval in the experiment (only it's longer or shorter).
* The motivation behind including time-limits was as follows: Time limits have been shown to affect risk taking and moderate the effects of framing in previous studies including the one that we replicated in the current study. Stephens risk sensitivity theory provides account for the sequential component of risk behavior when need-thresholds are involved (H10).
We emphasized the motivation of including time limits in the manuscript.

As far as I can tell, the authors run regressions treating each subject-trial as one observation. Observations coming from the same individual are obviously not independent. Strictly speaking, there are thus only 27 independent observations. For example, at least 20 out of 27 subjects would have to make more risk-seeking choices in on framing than the other for the p value to be below 0.5.

*We used a psychophysical data-collection approach which is a common method in cognitive science (see, e.g., (De Martino et al., 2006, Science; Guo et al., 2017, Psychological Science; Diederich & Trueblood, 2018, Psychological Review; Diederich et al., 2020, Theory and Decision; Wyszynski et al., 2020, PLOS ONE; Roberts et al., 2021, Psychological Science). Instead of presenting few trials to many participants as it is typically done in social science, fewer participants perform many more trials here.
As commonly practiced, we determined the sample size according to a power analysis based on established parameters, the smallest effect size of an effect observed in previous work (Need 3600 x Frame; Diederich, 2020), and the statistical method we used for the analysis. In particular, we used generalized linear mixed model analysis. We considered the variability in responses of participants to different trials by including the participants and the trials as random effects in the regression model (see e.g., Judd et al., 2017, Annual Review of Psychology; and Baayen et al., 2008; Journal of Memory and Language, for details). However, we performed another power analysis using a higher required power and increased our sample size accordingly.

My understanding is that the "needs" were payoff-irrelevant. How were they induced? I don't know because the instructions are not provided.
* Thanks for pointing this out. We added a sentence that should clarify the relationship between the need and the payoff in the materials section. And we uploaded the experiment instructions for participants to osf.io.

Minor issues:

p. 2 "Unusual disease" – It's customary to refer to it as "Asian disease" instead. Is politically incorrect now? I can't see how the use of this term implies anything wrong about the Asians. I understand the reasons not to name real diseases after geographic locations https://www.nature.com/articles/s41588-020-0617-2 but none of them applies to an abstract, fictitious Asian Disease considered by T&K.
* Irrespective from whether or not the terminus is "politically incorrect", we not want to label a disease which is expected to kill people with any region of origin. As the authors of the article that you have suggested indicate, naming infectious diseases after a location stigmatizes entire areas and ethnicities. This is something we would like to avoid. Relabeling the Asian disease problem in "unusual" disease problem have also been done previously in recent work (e.g., Wall et al., 2020, Judgement and decision making; Mayiwar and Björklund, 2021, frontiers in psychology; Levin et al., 2021, Positive Psychology and Disability Series book series).
To avoid confusion, we added the reference to the "Asian Disease Problem" to the footnote.

p. 6 "80 points were initial taken from the budget" -- ? should it be "initially"? anyway, I am not sure it's the optimal term. It suggests that even more point are taken from the budget subsequently (in the same trial) which is not the case.
*Thanks for noting this. We rephrased this sentence

Figure 1 is confusing. Why do panels A and B have the same label? Same for panels C and D.
* A and B are examples for a gain (A) and a loss (B) trial as presented in the study of Diederich et al. 2020. C and D are examples for a gain (C) and a loss (D) trial as presented in the current study. We emphasized the panel labels in the captions of figure 1 and figure 2.

Reviewer #2 comments:

(1) I am somewhat hesitant about using foraging behavior and the Stone-Geary utility framework to motivate some aspects of the study and H10. With foraging, there is an element of exploration, actual consumption, and/or energy expenditure that are absent in the study design. Similarly, the main premise that seems to be utilized about the Stone-Geary utility framework is the idea that consumers have a baseline level of consumption for goods that needs to be met before the utility of that good is influenced by consumer preferences.

* We agree that our study design does not directly correspond to foraging behavior. However, the energy budget rule has been successfully adopted and generalized to human decision making under risk (see the work of S. Mishra and colleagues). Thus, it is one of two theories that have been used to explain human risk-taking behavior in the context of need. The other model is the Stone-Geary Utility Framework (see Diederich et al., 2020) which discusses the role of need in expenditure decisions, analogous to those examined in the current study. The current study attempts to bring parts of both theories together to contribute to a better understanding of the impact of need-levels in risky decisions about losses and gains. We have made changes in the corresponding paragraphs of the introduction to make this point clearer.

The application of the Stone-Geary utility framework does seem to have a direct 1:1 correspondence with the present study design. Instead, based on the methods description, it seems that 'need' is manipulated based on the goal or target amount participants are told to aim for. If this is the case, it seems that there is a relationship between need and goal/goal setting. The description of need in the manuscript is difficult to grasp in the context of the present study. A more concrete connection to the present' study methodology and connection to goals/goal setting could make this clearer. The paper cited below is an example of goal setting and risk-taking that seems to align well with the present study design.

Schiebener, J., Wegmann, E., Pawlikowski, M., & Brand, M. (2014). Effects of goals on decisions under risk conditions: Goals can help to make better choices, but relatively high goals increase risk-taking. Journal of Cognitive Psychology, 26(4), 473-485.

* Thank you very much for this suggestion. We included relevant literature on goals in risky decision making in our manuscript.

Sample size & Power analysis: The sample size seems extremely small.
Please state the effect sizes observed in Diederich et al. that were used in the power analysis. What hypotheses were the power analyses conducted for? What statistical analysis was the power analysis modeled off of (e.g., t-test, correlations, regression, etc)? While the within-subjects design is a strength of the study, a larger sample size or replication study would strengthen the conclusions drawn from study.

* We used a psychophysical data-collection approach which is a common method in cognitive science. (see, e.g., (De Martino et al., 2006, Science; Guo et al., 2017, Psychological Science; Diederich & Trueblood, 2018, Psychological Review; Diederich et al., 2020, Theory and Decision; Wyszynski et al., 2020, PLOS ONE; Roberts et al., 2021, Psychological Science. Instead of presenting few trials to many participants as it is typically done in social science, fewer participants perform many more trials here.
As commonly practiced, we determined the sample size according to a power analysis based on established parameters, the smallest effect size of an effect observed in previous work (Need 3600 x Frame; Diederich, 2020), and the statistical method we used for the analysis. In particular, we used generalized linear mixed model analysis. We considered the variability in responses of participants to different trials by including the participants and the trials as random effects in the regression model (see e.g., Judd et al., 2017, Annual Review of Psychology; and Baayen et al., 2008; Journal of Memory and Language, for details). However, we performed an additional power analysis using higher required power and increased our sample size accordingly.
We have added the missing information to our manuscript.

(3) "Finally, we included two response time limits: 1 second or 3 seconds for a given block of trials." How was this time limit calculated? In other words, what is the scientific evidence to show that 1 and 3 seconds are appropriate time limits? Was there a pilot test to determine average response time, and time limits were adjusted from there? Or, something else? I can see that these are the time limits used in the authors' previous paper (Diederich et al., 2020), but the evidence for these time limits is not specified in this previous paper either.

* The exact values of time limits were taken from previous studies investigating the effect of time limits on risky choice using similar designs (Guo et al., 2017, Psychological Science; Diedrich et al., 2018, Judgement and decision making). The determination of long time-limits (3s) goes back to the mean response time plus the

standard deviation observed in pilot studies which not included any time-limits. We have added this information to our manuscript.

(4) Results: were outliers screened for in the Figure 3 analysis? Visually, it looks like there may be an outlier in the decisions about gains graph.
* Thank you for pointing this out. We screened for outliers by using the Rosner's test and identified one outlier. We have added this information to our manuscript.

Minor:
The idea of calling the initial amount of points a budget is a bit of a stretch. Budgeting with low-stakes points does not seem to have sufficient external validity to truly call it a budget.
* The personal budget and the amounts initially taken are two different things. We have modified the relevant sections of the manuscript to more clearly distinguish between the personal budget and the initial amount taken from the budget for each trial.

"Responses to a triple were collapsed for evaluation." I don't understand what this means.
* The values of Amounts '20', '40', '60', and '80' flanked by plus/minus one, resulting in the following set of four triples: '19,20,21'; '39,40,41'; '59,60,61'; and '79,80,81'). The responses to each triple were collapsed for evaluation, i.e., the three amounts of a triple were treated as one basic point amount. We rephrased the corresponding paragraph.

The level of detail in the Apparatus section isn't essential to understanding the study given that figures are provided.
*We agree and replaced this section with a shorter version. We moved the original version of the apparatus to the supplementary material.

In general, the methods are difficult to follow and could be improved with better organization and distinctive subheadings for each manipulation (frames, time limits, need levels, etc).
*We reorganized this section by adding subsections.

Discussion: a conclusion paragraph would be helpful
*We added concluding remarks to the summary and discussion section.
